# Progressive High-Grade Atrioventricular Block in a Young Adult: Distinguishing Reversible Causes From Lev-Lenègre Disease

**DOI:** 10.7759/cureus.108608

**Published:** 2026-05-10

**Authors:** Emanuel Zayas Diaz, Amarjit Kaur Sekhon, Hector Vega, Genesis J Mojica Sanchez, Shrilkumar Bhatt

**Affiliations:** 1 Internal Medicine, Abrazo Health System, Goodyear, USA; 2 Internal Medicine, Universidad Autonoma de Guadalajara, Guadalajara, MEX

**Keywords:** atrioventricular block, bifascicular block, bradyarrhythmia, conduction system disease, his-purkinje system, leadless pacemaker, lev-lenegre disease

## Abstract

High-grade atrioventricular (AV) block in young adults is uncommon and poses a diagnostic challenge when distinguishing reversible causes from intrinsic conduction system disease. We present a 27-year-old previously healthy male who presented with dizziness and was found to have a complete heart block with a wide QRS escape rhythm requiring temporary pacing. Initial evaluation revealed metabolic abnormalities and positive Coxsackie serology; however, correction of these factors did not result in improvement of conduction. Echocardiography demonstrated a normal cardiac structure. Given the possibility of a reversible etiology, permanent pacing was initially deferred. Two months later, the patient re-presented with near-syncope and progression to bifascicular block with first-degree AV block, consistent with progressive His-Purkinje system disease. He subsequently underwent successful dual-chamber leadless pacemaker implantation with resolution of symptoms. This case highlights the importance of recognizing progressive conduction system disease, such as Lev-Lenègre disease, even in young patients with apparent reversible factors, and emphasizes the role of serial electrocardiographic evaluation in guiding timely management.

## Introduction

Atrioventricular (AV) block refers to delayed or interrupted conduction between the atria and ventricles. Clinically, the level of block is important because nodal disease is often more stable and potentially reversible, whereas infranodal disease (high-grade AV block) involving the His-Purkinje system is more likely to progress and require permanent pacing.

High-grade AV block in young adults is uncommon [1] and often signals an underlying systemic or potentially reversible process. Although most commonly associated with age-related degenerative fibrosis of the conduction system, its occurrence in younger patients suggests an alternative etiology, warranting thorough evaluation.

In this population, high-grade AV block is frequently linked to metabolic derangements, viral myocarditis [2], or infectious etiologies, including Lyme disease. Identification of these reversible contributors is essential, as timely treatment may restore normal conduction and obviate the need for permanent pacing. However, clinical differentiation between transient conduction abnormalities and early manifestations of intrinsic conduction system disease remains challenging.

This uncertainty creates a management dilemma regarding the timing of permanent pacemaker implantation, particularly when initial evaluation suggests a reversible etiology but clinical progression remains unpredictable.

A key but often underrecognized cause is Lev-Lenègre disease, an idiopathic progressive fibrosis of the His-Purkinje system that classically is reported in older adults, although rare early-onset presentations have been described [3]. In such cases, conduction disease may evolve despite initial stabilization, ultimately necessitating permanent device therapy.

We present a case of high-grade AV block in a young adult in whom initially suspected reversible etiologies failed to account for persistent conduction abnormalities, ultimately revealing an evolving intrinsic conduction system disease requiring permanent pacing.

## Case presentation

A 27-year-old previously healthy male presented to the emergency department with progressive dizziness and lightheadedness. He denied chest pain, syncope, dyspnea, fever, recent viral illness, or substance use. There was no family history of sudden cardiac death, cardiomyopathy, or known conduction system disease.

On initial evaluation, the patient was found to be bradycardic, and electrocardiography demonstrated complete AV block with AV dissociation and a wide QRS escape rhythm (Figure 1). Given the high-grade conduction abnormality and risk of hemodynamic instability, the patient was admitted to the intensive care unit and underwent placement of a temporary transvenous pacemaker.

**Figure 1:**
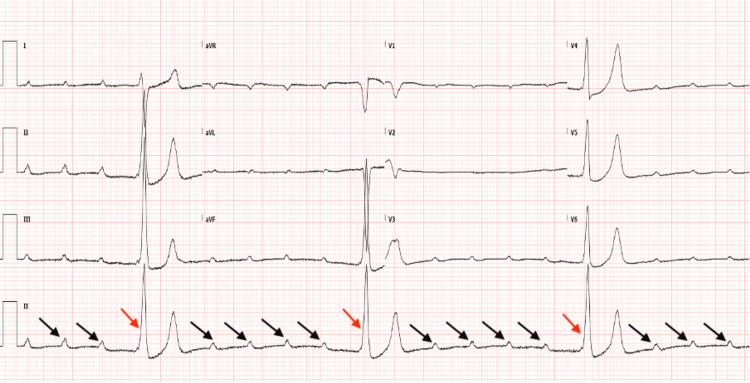
Initial electrocardiogram demonstrating complete atrioventricular block. Arrows indicate independent P waves, atrioventricular dissociation, and a wide QRS escape rhythm, supporting an infranodal pattern of conduction disease. Black arrows indicate independent P waves. Red arrows indicate a wide QRS escape rhythm.

Transthoracic echocardiography revealed normal cardiac structure and preserved left ventricular systolic function with an estimated ejection fraction of 55-60%, without evidence of valvular disease or regional wall motion abnormalities.

Initial laboratory evaluation was notable for acute kidney injury and hyperkalemia, both of which were corrected with appropriate medical management. However, resolution of these abnormalities did not improve the patient’s conduction abnormalities. Thyroid studies demonstrated elevated thyroid-stimulating hormone (TSH) and mildly elevated thyroxine (T4) levels, with a normal free triiodothyronine (T3) level (possible lab error). Thyroid ultrasound showed a homogeneous gland without nodules or structural abnormalities.

An extensive infectious and autoimmune workup was performed. Viral serologies were notable for Coxsackie A24, B1, B3, B4, B5, and B6 positivity, suggestive of prior exposure, but having in mind that positive serology alone does not establish active myocarditis and lacks specificity for causation. Autoimmune testing, including rheumatoid factor, anti-cyclic citrullinated peptide antibodies, anti-double-stranded DNA antibodies, thyroid peroxidase antibodies, and thyroglobulin antibodies, was negative (Table 1).

**Table 1:** Laboratory and Serologic Evaluation for Reversible Causes of High-Grade AV Block Coxsackie titers at 1:100 indicate low-titer seropositivity or prior exposure, depending on laboratory interpretation. Negative Coxsackie IgM results and absence of myocardial dysfunction make active viral myocarditis less likely.

Category	Test	Result	Reference Range/Normal Value	Clinical Interpretation
Infectious	HIV antibody/P24 antigen screen	Nonreactive	Nonreactive	No evidence of HIV infection
Infectious	Coccidioides IgG	Negative	Negative	No serologic evidence of prior or active coccidioidomycosis
Infectious	Coccidioides IgM	Negative	Negative	No evidence of acute coccidioidomycosis
Infectious	Lyme disease PCR	Negative	Negative	No evidence of Lyme disease by PCR
Infectious	Lyme total antibody	Negative	Negative	No serologic evidence of Lyme disease
Viral serology	Coxsackie A	Negative	Negative	Negative screening result
Viral serology	Coxsackie A7	Negative	Negative	No evidence of Coxsackie A7 exposure
Viral serology	Coxsackie A16	Negative	Negative	No evidence of Coxsackie A16 exposure
Viral serology	Coxsackie A24	1:100	<1:100	Low-titer/borderline positive; suggests prior exposure but does not confirm active myocarditis
Viral serology	Coxsackie B1	1:100	<1:100	Low-titer/borderline positive; suggests prior exposure
Viral serology	Coxsackie B2	Negative	Negative	No evidence of Coxsackie B2 exposure
Viral serology	Coxsackie B3	1:100	<1:100	Low-titer/borderline positive; suggests prior exposure
Viral serology	Coxsackie B4	1:100	<1:100	Low-titer/borderline positive; suggests prior exposure
Viral serology	Coxsackie B5	1:100	<1:100	Low-titer/borderline positive; suggests prior exposure
Viral serology	Coxsackie B6	1:100	<1:100	Low-titer/borderline positive; suggests prior exposure
Viral serology	Coxsackie A7 IgM	Negative	Negative	No evidence of acute Coxsackie A7 infection
Viral serology	Coxsackie A9 IgM	Negative	Negative	No evidence of acute Coxsackie A9 infection
Viral serology	Coxsackie A16 IgM	Negative	Negative	No evidence of acute Coxsackie A16 infection
Viral serology	Coxsackie A24 IgM	Negative	Negative	No evidence of acute Coxsackie A24 infection
Autoimmune	ANA direct	Negative	Negative	No evidence of systemic autoimmune disease by ANA screening
Autoimmune	Double-stranded DNA antibody	1	0–9	Negative; argues against systemic lupus erythematosus
Autoimmune	CCP IgG/IgA	Negative	0–19	Negative; argues against rheumatoid arthritis
Autoimmune	Rheumatoid factor	12.9	<20	Negative
Autoimmune	Sjögren’s anti-SSA	<0.2	<0.2	Negative
Autoimmune	Sjögren’s anti-SSB	<0.2	<0.2	Negative
Autoimmune	Anti-Ro	Negative	Negative	Negative
Autoimmune	Anti-La	Negative	Negative	Negative
Thyroid	TSH	7.90	0.45–5.33	Elevated
Thyroid	Free T4	1.86	0.61–1.12	Elevated
Thyroid	Free T3	3.90	2.50–3.90	Upper limit of normal
Thyroid antibodies	Thyroid peroxidase antibody	13	0–34	Negative
Thyroid antibodies	Thyroglobulin antibody	<1	0.0–0.9	Borderline/near negative, depending on laboratory interpretation
Inflammatory marker	C-reactive protein, quantitative	132	<10	Elevated inflammatory marker; nonspecific and not diagnostic of myocarditis

During hospitalization, the patient continued to exhibit sinus pauses and high-grade AV conduction abnormalities. After removal of the temporary pacing wire, he demonstrated a persistent second-degree AV block with intermittent pauses. Given the patient’s young age, absence of structural heart disease, and concern for a potentially reversible etiology such as myocarditis, a shared decision was made to defer permanent pacemaker implantation. The patient was discharged in stable condition with plans for outpatient cardiac magnetic resonance imaging, ambulatory cardiac monitoring, and close cardiology follow-up. The patient visited the cardiologist office one month after this hospitalization, and an MRI was planned but not completed.

Approximately two months later, the patient was admitted for near-syncope. Repeat electrocardiography demonstrated sinus rhythm with first-degree AV block, right bundle branch block, and left posterior fascicular block, consistent with bifascicular conduction system disease (Figure 2). Given the progression of conduction abnormalities and recurrence of symptoms, the patient underwent elective dual-chamber leadless pacemaker implantation without complications.

**Figure 2:**
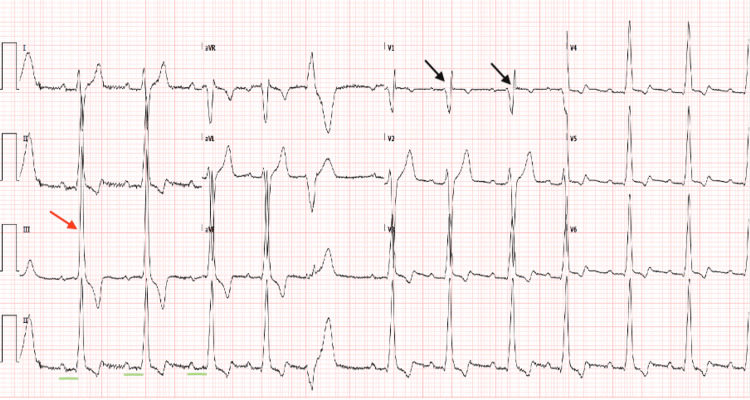
Follow-up electrocardiogram demonstrating sinus rhythm with first-degree atrioventricular block, right bundle branch block, and left posterior fascicular block. Arrows highlight the prolonged PR interval, right bundle branch block morphology, and features consistent with bifascicular conduction disease. Black arrows indicate a right bundle branch block pattern (V1). The red arrow indicates right axis deviation (LPFB). Green lines indicate prolonged PR intervals.

Post-procedure chest imaging confirmed appropriate device positioning (Figure 3), and a follow-up echocardiogram demonstrated no pericardial effusion. The patient remained hemodynamically stable and reported complete resolution of symptoms. He was discharged home with outpatient follow-up and continued to do well without recurrence of dizziness or pre-syncope (Table 2).

**Figure 3:**
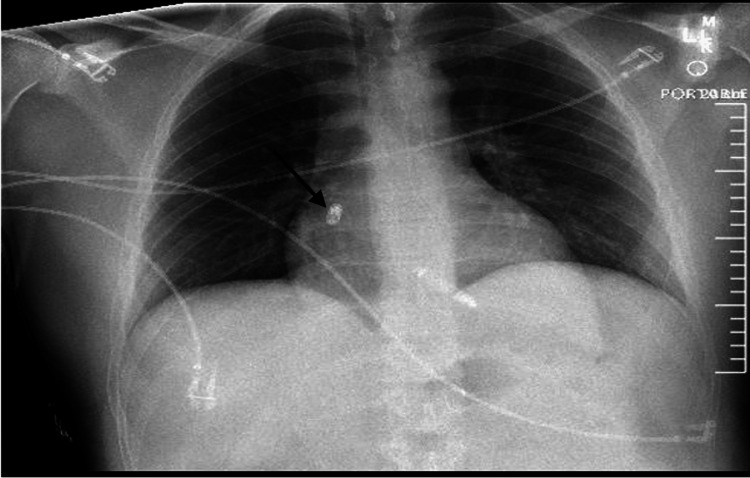
Post-procedure chest radiograph demonstrating appropriate leadless pacemaker positioning. The arrow indicates the intracardiac leadless pacemaker device.

**Table 2:** Clinical Timeline and Evolution of Conduction Abnormalities AV: atrioventricular; RBBB: right bundle branch block; LPFB: left posterior fascicle block

Time point	Key Findings	Management Decision
Initial presentation	Dizziness, lightheadedness, complete AV block with wide QRS escape rhythm	ICU admission and temporary transvenous pacing
Hospital course	Persistent pauses and second-degree AV block type I after temporary wire removal	Permanent pacing discussed but deferred due to possible reversibility and patient preferences
Discharge	Stable condition with persistent concern for conduction disease	Ambulatory monitoring, plan for outpatient cardiac MRI, cardiology follow-up
Two-month follow-up	Near-syncope with first-degree AV block, RBBB, and LPFB	Permanent pacing pursued
Post-procedure	Dual-chamber leadless pacemaker placed without complications	Symptom resolution

## Discussion

AV block represents a spectrum of conduction abnormalities, classified into first-, second-, and third-degree blocks. These can also be categorized based on their location as nodal (above the His bundle) or infranodal (at or below the His-Purkinje system). Clinically, this distinction is critical, as it helps predict both reversibility and risk of progression [4].

First-degree AV block is defined by a prolonged PR interval, reflecting delayed conduction between the atria and ventricles, and is typically nodal. Second-degree AV block involves intermittent failure of conduction and is commonly divided into Mobitz type I and Mobitz type II. Mobitz type I is usually nodal, whereas Mobitz type II is typically infranodal and more concerning. Third-degree AV block represents complete AV dissociation and can occur at either the nodal or infranodal level.

From a clinical standpoint, identifying whether a block is nodal or infranodal is essential. Nodal blocks are often associated with reversible causes and tend to be more stable, particularly when the escape rhythm is narrow. In contrast, infranodal blocks are more likely to be irreversible, may progress unpredictably, and often require permanent pacemaker implantation. The width of the QRS complex is particularly helpful in this distinction, as a wide QRS escape rhythm suggests involvement of the His-Purkinje system and is associated with less stable conduction [5].

In this case, an important diagnostic consideration was differentiating between myocarditis and progressive conduction system disease. Myocarditis is an inflammatory condition of the myocardium that is typically acute in onset and may be associated with infections, autoimmune conditions, or drug exposure [2]. Although it can present with conduction abnormalities, it is often accompanied by myocardial dysfunction and tends to improve over time. In contrast, progressive conduction disease, such as Lev-Lenègre disease, is characterized by fibrosis of the His-Purkinje system and typically follows a stepwise ECG progression in the absence of structural heart disease.

Several features in this case favor progressive conduction system disease over myocarditis. First, the initial ECG demonstrated a wide QRS escape rhythm, suggesting infranodal involvement from the outset. Second, the patient exhibited a progressive pattern of conduction abnormalities over time, evolving from complete heart block to bifascicular block with first-degree AV block. This stepwise deterioration is more consistent with degenerative conduction disease than with transient inflammatory processes [3]. Additionally, there was no evidence of myocardial involvement, as the echocardiogram remained normal and there were no clinical signs of myocarditis. The timeline also argues against myocarditis, as conduction abnormalities typically improve rather than worsen over weeks to months. Although viral serology suggested prior exposure, this alone is insufficient to establish a diagnosis of active myocarditis.

During the patient’s initial hospitalization, permanent pacemaker implantation was deferred. This decision was reasonable given the patient’s young age, partial recovery of AV conduction, and the possibility of a reversible etiology. Shared decision-making also played a role, as the patient preferred to defer intervention at that time. However, upon re-presentation with near-syncope and progression to bifascicular block, the indication for permanent pacing became clear.

Current guidelines support permanent pacemaker implantation in patients with symptomatic high-grade AV block and in those with infranodal conduction disease due to the risk of progression [4]. In this case, ECG findings were central to risk stratification and guided management decisions at both presentations.

Long-term device-related complications are particularly relevant in younger patients because they may require pacing for several decades. Traditional transvenous systems carry risks of lead fracture, lead infection, venous stenosis or occlusion, pocket complications, and future lead extraction or revision. These cumulative risks are important when selecting a device strategy in younger individuals expected to require lifelong pacing. Prior studies have highlighted the long-term implications of pacemaker therapy in younger adults and the procedural complexity associated with transvenous lead extraction [6]. A leadless pacemaker was ultimately selected for this patient. These devices offer several advantages, particularly in younger individuals, including the absence of transvenous leads, reduced risk of infection, and avoidance of long-term vascular complications [7]. Additionally, they are associated with shorter recovery times and fewer activity restrictions, making them an attractive option in appropriately selected patients.

The unique contribution of this case is the longitudinal demonstration of evolving conduction disease in a young patient initially suspected to have a reversible cause of high-grade AV block. The initial presentation with a complete AV block and a wide QRS escape rhythm suggested infranodal involvement, but the patient’s young age and possible reversible factors appropriately led to conservative management. The later progression to bifascicular conduction disease with first-degree AV block and recurrent symptoms provided a clear temporal pattern supporting progressive His-Purkinje system disease. This case emphasizes that apparent transient improvement should not be interpreted as definitive recovery, particularly when the initial ECG suggests infranodal disease.

## Conclusions

AV block in young adults represents a significant diagnostic and management challenge, particularly when differentiating reversible causes from progressive conduction system disease. This case demonstrates that apparent reversible factors, including metabolic abnormalities or positive viral serology, should not lead clinicians to assume complete reversibility when ECG findings suggest infranodal disease. Serial electrocardiographic evaluation was central to this case. The transition from complete heart block with a wide QRS escape rhythm to bifascicular conduction disease with first-degree AV block supported likely progressive His-Purkinje system involvement. Although definitive exclusion of myocarditis or infiltrative disease was limited by the absence of a completed cardiac MRI or biopsy, the clinical course favored progressive conduction system disease over a transient inflammatory process.

Close follow-up is particularly important when permanent pacing is initially deferred. Ultimately, progression of conduction abnormalities and recurrence of symptoms should prompt reconsideration of device therapy. Leadless pacemaker systems represent a promising option in appropriately selected young patients, offering effective symptom control while reducing long-term complications associated with traditional transvenous pacing.
